# Psychosocial determinants of anti-VEGF treatment adherence in AMD patients: optimization of one-stop intravitreal injection service model

**DOI:** 10.3389/fmed.2026.1745453

**Published:** 2026-04-10

**Authors:** Xi Zhang, Bingjie Cui, Yingyue Liu, Xiangning Ji, Xiaoyu Tian, Siqing Hou, Lidong Yang, Junshu Yang

**Affiliations:** 1 Ophthalmology Medical Center, Cangzhou Central Hospital, Cangzhou, Hebei, China; 2 Cangzhou Eye Hospital, Cangzhou, Hebei, China; 3 Hebei Medical University, Shijiazhuang, Hebei, China

**Keywords:** anti-VEGF treatment, artificial intelligence, neovascular age-related macular degeneration, one-stop service, psychosocial factors, treatment adherence

## Abstract

**Objective:**

To investigate modifiable psychosocial determinants of anti-VEGF treatment adherence in patients with neovascular age-related macular degeneration (nAMD) and evaluate the optimization effects of a one-stop intravitreal injection service model.

**Methods:**

This historical control mixed-methods study included patients receiving anti-VEGF treatment at Cangzhou Regional Ophthalmology Center from August 2022 to October 2024. Patients were divided into three groups based on service models: traditional multiple-visit group (historical control, *n* = 165), one-stop standard group (*n* = 161), and one-stop AI-enhanced group (*n* = 162). The Hospital Anxiety and Depression Scale (HADS) was used to assess psychological status, and geographic information systems analyzed spatial accessibility impacts. Primary outcome measures included 12-month retention rate, early discontinuation rate (within 6 months), and appointment adherence rate. In-depth semi-structured interviews were conducted with patients from the one-stop standard and AI-enhanced groups, using thematic analysis to identify key influencing factors. Logistic regression analysis was used to analyze adherence predictors.

**Results:**

Compared to the historical control group, the one-stop standard and AI-enhanced groups showed significantly reduced clinic-to-injection time (23.87 vs. 6.47 vs. 6.01 h, *P* < 0.05), significantly improved 12-month retention rates (52.12 vs. 73.29 vs. 85.80%, *P* < 0.05), and significantly reduced early discontinuation rates (29.09 vs. 17.39 vs. 9.88%, *P* < 0.05). Regarding clinical outcomes, patients in the AI-enhanced group showed more significant best-corrected visual acuity (BCVA) improvement (logMAR change: −0.08 vs. −0.12 vs. −0.17, *P* < 0.05) and more pronounced central retinal thickness reduction (57.83 vs. 86.92 vs. 111.75 μm, *P* < 0.05). Multifactorial analysis showed that residential distance >38 km, baseline high anxiety levels, and baseline depressive symptoms were independent risk factors for treatment discontinuation. AI-enhanced intervention significantly reduced early discontinuation risk (*P* < 0.05). Qualitative analysis identified five main themes: treatment anxiety, service experience, expectation management, social support, and service improvement needs. Safety event incidence rates showed no significant differences between groups (*P* > 0.05).

**Conclusion:**

The one-stop intravitreal injection service model significantly improved treatment adherence in nAMD patients, with AI-enhanced intervention further optimizing outcomes. Baseline anxiety and depression levels, along with geographic distance, are important modifiable determinants of treatment adherence. Personalized service models integrating psychosocial interventions provide new insights for precision management of chronic eye diseases.

## Highlights

- Maladaptive expectations and anxiety impact anti-VEGF therapy adherence in nAMD
- Geographic distance from service centers increases treatment discontinuation risk
- AI-enhanced one-stop service boosts retention, reduces injection intervals in nAMD

## 1 Introduction

Neovascular age-related macular degeneration (nAMD) is a leading cause of irreversible vision loss worldwide, affecting millions of older adults (1, 2). Anti-vascular endothelial growth factor (anti-VEGF) injections have transformed the treatment landscape of nAMD, improving visual acuity outcomes and preventing disease progression (3–5). However, real-world data suggest that long-term success is often limited by suboptimal adherence (6). Incomplete treatment persistence intervals may severely compromise therapeutic efficacy (7, 8).

A growing body of literature has identified psychosocial elements—such as patient perceptions of disease severity, treatment-related anxiety, and the complexity of healthcare logistics—as key factors influencing adherence (9–11). Patients with unrealistic or maladaptive expectations, for instance, are at higher risk for disengagement when faced with the chronic, often demanding nature of repeated intravitreal injections (12). Moreover, operational barriers—long waiting times, high direct and indirect costs, and travel difficulties—compound these issues in resource-limited settings (13–15). Given the potentially large burden on both patients and healthcare systems, the quest for a streamlined and patient-centered care model is paramount.

One-stop intravitreal injection services have gradually been implemented in some tertiary referral centers. This service model aims to reduce patient waiting times and improve treatment adherence by consolidating pre-injection assessments and related procedures into a single visit (16). Furthermore, with the continuous advancement of technology, Artificial Intelligence (AI)-driven interventions have shown great potential in optimizing treatment processes. Through precise data analysis and predictive models, AI can develop personalized treatment plans tailored to individual patients, further improving treatment outcomes and enhancing adherence (17).

Current research on factors influencing anti-VEGF treatment adherence in nAMD patients has not fully considered the systematic analysis of interactions between psychosocial factors and service models (18). Additionally, application research of AI technology in chronic ophthalmic disease management remains relatively limited, and its specific mechanisms and effects on adherence improvement require further validation. This study is a historical control, mixed-methods study conducted at the Cangzhou Regional Ophthalmology Center in China, aiming to identify modifiable psychosocial determinants of anti-VEGF adherence in newly implemented one-stop intravitreal injection models. By comparing three service models—traditional multiple-visit model, one-stop standard model, and one-stop AI-enhanced model—this study hopes to provide evidence-based support for precision management of nAMD patients and offer experiences that can be applied to service optimization for other chronic eye diseases.

## 2 Methods

### 2.1 Study design and setting

This study employed a historical control, prospective mixed-methods design to evaluate a healthcare service delivery optimization initiative at the Department of Ophthalmology, Cangzhou Regional Ophthalmology Center. This was a quality improvement study comparing different clinical service models, not a trial of therapeutic interventions, as all patients received identical standard-of-care anti-VEGF treatment regardless of group assignment. The study included three distinct phases: a historical control phase using traditional visit models (August 2022 to July 2023), a one-stop standard model phase (August 2023 to February 2024), and a one-stop AI-enhanced model phase (March 2024 to October 2024). The study adhered to the principles of the Declaration of Helsinki (19).

### 2.2 Participants and recruitment

A total of 180 consecutive patients who received anti-VEGF treatment under the traditional multiple-visit model from August 2022 to July 2023 were retrospectively included as the historical control group. From August 2023 to October 2024, 358 consecutive patients newly diagnosed with nAMD and starting anti-VEGF treatment were prospectively recruited. Among these, 182 patients were in the one-stop standard model group (August 2023 to February 2024), and 176 patients were in the one-stop AI-enhanced model group (March 2024 to October 2024).

**Figure 1:**
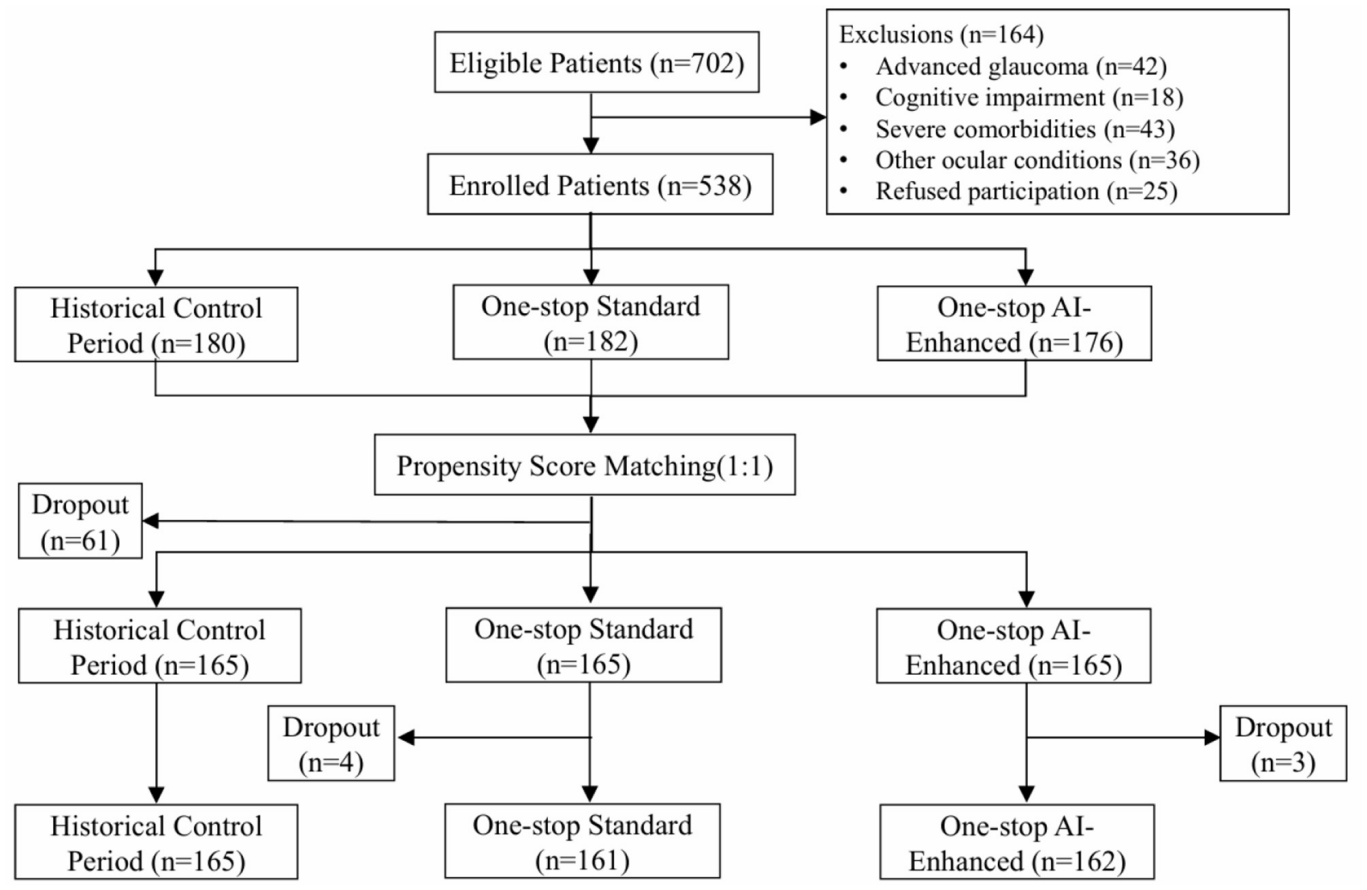
Patient screening and grouping flowchart.

After propensity score matching (PSM), the patient numbers in all three groups were adjusted to 165 patients each. After 12-month follow-up, the specific patient numbers were: one-stop standard model group 161 patients; one-stop AI-enhanced model group 162 patients. The one-stop standard model group lost four patients: one death from acute myocardial infarction, one death from stroke, and two patients who could not continue ophthalmic follow-up after malignant tumor diagnosis and chemotherapy. The one-stop AI-enhanced model group lost three patients: one death from acute myocardial infarction, one from accidental death, and one who could not continue ophthalmic follow-up due to severe infection. The patient research process is shown in Figure 1.

#### 2.2.1 Inclusion criteria

Age ≥50 years; confirmed presence of active choroidal neovascularization (CNV) through optical coherence tomography (OCT) and/or fluorescein angiography (FA); first diagnosis of nAMD or previous anti-VEGF treatment ≤ 3 times; willingness to comply with at least 12 months of follow-up; normal cognitive function able to understand and sign informed consent; basic Chinese reading and writing ability to complete questionnaires.

#### 2.2.2 Exclusion criteria

Presence of ocular diseases that may confound visual acuity assessment (such as advanced glaucoma or diabetic retinopathy); severe cognitive dysfunction hindering informed consent; inability to attend scheduled follow-up due to severe systemic comorbidities; expected survival <12 months; history of severe mental illness; participation in other clinical trials.

### 2.3 Treatment protocol

All patients received the same anti-VEGF treatment regimen:

#### 2.3.1 Drug selection and dosage

All patients received ranibizumab (Ranibizumab, Novartis Pharmaceuticals) intravitreal injection therapy at a standard dose of 0.5 mg/0.05 ml.

#### 2.3.2 Treatment regimen

Initial treatment phase: three consecutive monthly injections. Maintenance treatment phase: injection intervals were adjusted based on disease activity, with active disease maintaining 4-week intervals, stable patients extending to 6–8 weeks, with a maximum of 12 weeks. Disease activity assessment criteria included: BCVA decline ≥5 letters, new or persistent subretinal/intraretinal fluid, macular hemorrhage, neovascularization, pigment epithelial detachment, and other comprehensive anatomical structural changes (20).

#### 2.3.3 Standardized injection procedure

All intravitreal injections were performed by the same retinal specialist with over 10 years of experience to ensure technical consistency. Injection procedures strictly followed aseptic operating standards: tropicamide mydriasis 30 min preoperatively, surface anesthesia with oxybuprocaine eye drops, surgical field disinfection with 5% povidone iodine, injection site selection 3.5–4.0 mm from the limbus at the pars plana, vertical needle insertion using a 30 G needle. Postoperative routine levofloxacin eye drops were administered for infection prevention.

#### 2.3.4 Follow-up monitoring

All patients followed the same follow-up schedule: routine examinations 1 day, 1 week, and 1 month post-injection, with subsequent scheduling according to the treat-and-extend (T&E) protocol. Each follow-up included BCVA measurement, intraocular pressure monitoring, slit-lamp examination, and OCT scanning. Fluorescein angiography (FA) or OCTA examinations were performed at baseline, 3, 6, and 12 months, with additional examinations as needed.

### 2.4 Service models

#### 2.4.1 Traditional multiple-visit model (historical control)

Before August 2023, patients required 1-day hospitalization for comprehensive ophthalmic examination (such as OCT, slit-lamp examination, and laboratory tests), staying overnight for intravitreal injection surgery the next day, with total hospitalization time approximately 24 h.

#### 2.4.2 One-stop intravitreal injection model (from August 2023)

Under the new one-stop model, patients completed all pre-injection clinical assessments and laboratory examinations in the morning and underwent injection surgery in the afternoon (all injections performed by a single physician, approximately 5 min per patient), discharged after 1 h of observation. This optimized workflow reduced total hospitalization time to 6–7 h, eliminating overnight stays and creating a true “day hospital” experience.

It should be noted that the following service models represent variations in healthcare delivery logistics only; the actual medical treatment (anti-VEGF injection with ranibizumab) remained identical across all groups.

### 2.5 AI-enhanced intervention protocol (from March 2024)

From March 2024, AI-enhanced intervention was introduced for all newly enrolled patients, comprising three comprehensive components:

#### 2.5.1 System overview

The AI intervention, named “nAMD Treatment Adherence Optimization AI System,” was developed in-house by our research team as an integrated software platform designed to optimize the intravitreal injection service workflow. The system was built on Python 3.9 and Scikit-learn 1.0.2 machine learning library.

#### 2.5.2 Real-time expectation correction module

This module utilized a BERT-based pre-trained natural language processing model to analyze physician-patient consultation dialogues, identify patient concerns and knowledge gaps, and deliver personalized educational content. The system included visual aids showing realistic treatment trajectories and interactive simulations of injection procedures to reduce procedural anxiety. Educational content was validated through expert review to ensure consistency with clinical guidelines.

#### 2.5.3 Adaptive scheduling algorithm

The core component was a gradient boosting decision tree model (implemented using XGBoost 1.6.0) to predict patients' appointment non-adherence risk and optimize scheduling. The model was trained on de-identified electronic medical record data from our center, encompassing 3 years (August 2018 to July 2021) of records from nAMD patients receiving anti-VEGF therapy. The training dataset comprised 8,742 visit records from 612 patients, with features including demographic characteristics, travel distance, historical attendance patterns, weather data, and baseline HADS anxiety/depression scores. Data preprocessing included handling missing values, outlier detection, and feature standardization. Internal validation using five-fold cross-validation demonstrated a mean AUC of 0.81 (95% CI: 0.78–0.84) for predicting appointment non-adherence risk. Patients with predicted non-adherence probability exceeding 0.3 were flagged for priority scheduling at preferred time slots and received additional reminder messages via SMS or WeChat at 48 and 24 h before appointments.

#### 2.5.4 Algorithmic bias mitigation

To prevent algorithmic bias, variables representing healthcare accessibility (residential distance, insurance type) were explicitly included to avoid systematic bias against patients from remote areas. Fairness analysis examining model performance across subgroups (education levels, residential distances) revealed no significant performance disparities.

#### 2.5.5 Community-based pre-treatment counseling

Community-Based Pre-treatment Counseling: This component was implemented through collaboration between our ophthalmology center and community health service centers within the Cangzhou region. Community health workers (CHWs) identified potential nAMD patients through multiple channels, including active screening during routine health check-ups for residents aged ≥50 years, referral coordination with primary care physicians, and follow-up outreach to patients who had previously discontinued treatment. For patients who presented directly to our hospital without prior community contact, a bidirectional referral system was implemented—after diagnosis confirmation, our center notified the relevant CHW based on patient residential information, and the CHW then conducted home visits to provide supplementary education and establish ongoing communication for appointment reminders. CHWs performed functions that complemented hospital staff, including pre-visit education in familiar community settings, family education to improve caregiver support, transportation coordination for elderly patients in rural areas, culturally appropriate communication in local dialects, and post-visit follow-up to reinforce treatment instructions and identify adherence barriers. CHWs received standardized training covering nAMD pathophysiology, anti-VEGF treatment principles, and patient communication skills.

#### 2.5.6 Human oversight

All AI-generated recommendations were reviewed by clinical staff before patient communication, ensuring human oversight throughout intervention delivery.

### 2.6 Data collection and measurements

#### 2.6.1 Baseline survey

Collection of demographic characteristics (age, gender, education level, medical insurance type, residential address, marital status) and disease-related information (disease duration, initial symptoms, previous treatment history, binocular vision, intraocular pressure, fundus conditions, comorbidities).

#### 2.6.2 Psychosocial assessment

Psychosocial assessment was conducted for prospective cohort patients (one-stop standard and AI-enhanced groups). Hospital Anxiety and Depression Scale (HADS): Patients were screened at baseline and 3-month intervals (3, 6, 9, and 12 months after treatment initiation). HADS generates independent subscale scores for anxiety (HADS-A) and depression (HADS-D). A cut-off value of ≥8 for each subscale indicates clinically significant symptoms (21).

#### 2.6.3 Geospatial accessibility

Geographic Information System (GIS) software (ArcGIS, Esri, Redlands, CA) was used to calculate road distance from patient home addresses to the hospital. Stratified analysis was performed based on the median distance of the study population.

#### 2.6.4 Clinic-to-injection time

Total time interval from patient registration to completion of intravitreal injection, automatically recorded through the hospital electronic medical record (EMR) system.

#### 2.6.5 12-month retention rate

Defined as the proportion of patients still receiving follow-up and treatment at 12 months after treatment initiation, including: (1) continuing planned anti-VEGF injection treatment; (2) temporarily discontinued injections but still attending scheduled follow-up monitoring; (3) patients with stable conditions for whom physicians recommended treatment suspension but continued regular follow-up. Excluded are cases lost to follow-up due to non-adherence factors such as death, severe systemic illness, or relocation.

#### 2.6.6 Early discontinuation rate

Defined as the proportion of patients who actively stopped treatment and no longer received any form of follow-up within 6 months of treatment initiation. Early discontinuation criteria: (1) patient actively requested treatment cessation; (2) continuous loss to follow-up exceeding 8 weeks with inability to contact; (3) explicit refusal to continue treatment or follow-up. Treatment termination due to objective factors such as death, systemic disease deterioration, or unavoidable relocation was not counted as early discontinuation.

#### 2.6.7 Appointment adherence rate

Actual visits/planned appointments × 100%

#### 2.6.8 Clinical outcome measures

Visual function assessment included best-corrected visual acuity (BCVA, logMAR values) and central retinal thickness (OCT measurement).

#### 2.6.9 Safety assessment

Included endophthalmitis incidence, intraocular pressure elevation, retinal detachment, and other serious adverse events.

#### 2.6.10 Qualitative data collection

Qualitative Data Collection: In-depth semi-structured interviews were conducted with a purposive sample of 42 patients (22 from the one-stop standard group and 20 from the AI-enhanced group), selected using maximum variation sampling to ensure diversity in age, gender, residential distance, treatment response, and adherence patterns. Interview domains included emotional reactions to treatment, perceived barriers, facilitating factor experiences, and service improvement suggestions. All interviews were conducted by two trained researchers (BC and YL), audio-recorded with participant consent, and transcribed verbatim within 48 h. Thematic analysis followed Braun and Clarke's six-phase framework. Two researchers independently coded the first 10 transcripts, achieving an inter-rater reliability (Cohen's kappa) of 0.83. Discrepancies were resolved through discussion until consensus was reached. The remaining transcripts were coded by one researcher with periodic cross-checking. Thematic saturation was determined when three consecutive interviews yielded no new themes, which occurred after 38 interviews; four additional interviews were conducted to confirm saturation. NVivo software (version 12, QSR International) was used to facilitate data organization and analysis (22).

### 2.7 Statistical analysis

All quantitative data were analyzed using R software (version 4.2.0). For comparisons between historical control and one-stop model groups, propensity score matching was used to adjust for baseline differences. The propensity score model included the following baseline variables: age, gender, education level (primary school and below/junior and senior high school/college and above), residential distance, medical insurance type (urban employee/rural cooperative and urban-rural resident), baseline best-corrected visual acuity (BCVA), central retinal thickness, and bilateral disease status. Nearest neighbor matching was used with a 1:1 matching ratio and caliper set at 0.2 standard deviations. Standardized mean difference (SMD) was used to assess matching balance after matching, with SMD <0.1 considered well-matched. Descriptive statistics were reported as mean ± standard deviation for continuous variables and frequency (percentage) for categorical variables. Normality of continuous variables was assessed using the Shapiro-Wilk test. Variables with *P* > 0.05 were considered normally distributed and analyzed using one-way ANOVA with Bonferroni *post-hoc* test; non-normally distributed variables (Shapiro-Wilk *P* < 0.05) were analyzed using the Kruskal-Wallis *H* test with Dunn's *post-hoc* test for pairwise comparisons. Categorical variables were analyzed using chi-square tests or Fisher's exact test. Changes in psychological status over time were analyzed using repeated measures analysis of variance. Univariate and multivariate logistic regression analyses were used to identify predictors of early discontinuation, with model goodness-of-fit assessed using the Hosmer-Lemeshow test. Geospatial analysis used ArcGIS software to calculate distances and perform stratified analysis. Qualitative data were analyzed using thematic analysis with NVivo software assistance. Missing data were handled using multiple imputation. Statistical significance was set at *P* < 0.05 (two-sided).

## 3 Results

### 3.1 Baseline characteristics

This study ultimately included 165 patients in the historical control group, 161 patients in the one-stop standard group, and 162 patients in the one-stop AI-enhanced group. All three groups showed SMD <0.1 for baseline characteristics including age, gender, education level, residential distance, medical insurance type, baseline visual acuity, and central retinal thickness, indicating good matching (Table 1).

**Table 1:** Comparison of baseline characteristics among three groups.

Characteristics	Historical control group (*n* = 165)	One-stop standard group (*n* = 161)	One-stop AI-enhanced group (*n* = 162)	SMD1	SMD2	SMD3
Demographics						
Age (years), mean ± SD	69.42 ± 8.17	70.08 ± 7.94	69.83 ± 8.51	0.081	0.049	−0.030
Female, *n* (%)	89 (53.94)	90 (55.90)	85 (52.47)	0.039	−0.029	−0.068
Education level, *n* (%)						
Primary school and below	78 (47.27)	80 (49.69)	74 (45.68)	0.049	−0.032	−0.081
Junior and senior high	71 (43.03)	66 (41.01)	72 (44.44)	−0.041	0.028	0.069
College and above	16 (9.70)	15 (9.32)	16 (9.88)	−0.013	0.006	0.019
Residential distance (km), median (IQR)	38.35 (24.20–53.80)	39.16 (25.45–53.23)	38.67 (24.38–52.92)	0.028	0.011	−0.017
Medical insurance type, *n* (%)						
Urban employee insurance	56 (33.94)	57 (35.40)	53 (32.72)	0.031	−0.026	−0.056
Rural cooperative/urban-rural resident insurance	109 (66.06)	104 (64.60)	109 (67.28)	−0.031	0.026	0.056
Clinical characteristics						
Baseline BCVA (logMAR), mean ± SD	0.68 ± 0.31	0.71 ± 0.29	0.69 ± 0.33	0.100	0.031	−0.063
Central retinal thickness (μm), mean ± SD	386.75 ± 94.28	390.92 ± 87.64	384.13 ± 96.85	0.046	−0.027	−0.072
Bilateral disease, *n* (%)	47 (28.48)	51 (31.68)	48 (29.63)	0.070	0.025	−0.042

Propensity score matching (1:1 ratio) was performed using nearest neighbor matching with a caliper of 0.2 standard deviations. Balance between groups was assessed using standardized mean differences (SMD), with SMD <0.1 indicating adequate balance. Continuous variables are presented as mean ± SD for normally distributed data or median (IQR) for skewed distributions; categorical variables are presented as n (%). SMD1, standardized mean difference between one-stop standard group vs. historical control group; SMD2, standardized mean difference between one-stop AI-enhanced group vs. historical control group; SMD3, standardized mean difference between one-stop AI-enhanced group vs. one-stop standard group; SD, standard deviation; IQR, interquartile range; BCVA, best-corrected visual acuity; logMAR, logarithm of the minimum angle of resolution.

### 3.2 Service model effectiveness comparison

Compared to the historical control group, the one-stop standard and AI-enhanced groups showed significantly reduced clinic-to-injection times of 6.47 and 6.01 h, respectively (*P* < 0.05). Appointment adherence rates improved from 76.38 to 84.17 and 91.35%. Most importantly, 12-month retention rates significantly increased from 52.12 to 73.29% (one-stop standard group) and 85.80% (AI-enhanced group). Early discontinuation rates within 6 months decreased significantly from 29.09% in the historical control group to 17.39% in the one-stop standard group and 9.88% in the AI-enhanced group (*P* < 0.05). Median BCVA improvements were −0.12 and −0.17 logMAR in the one-stop standard and AI-enhanced groups, respectively, both significantly better than −0.08 in the historical control group (*P* < 0.05). Similarly, reductions in central foveal thickness were greater, reaching 86.92 and 111.75 μm, compared to 57.83 μm in the control group (*P* < 0.05). Table 2 presents a comparison of operational indicators under the three service models.

**Table 2:** Comparison of operational indicators under different service models.

Indicator	Historical control group (*n* = 165)	One-stop standard group (*n* = 161)	One-stop AI-enhanced group (*n* = 162)	Test statistic	*P*-value
Clinic-to-injection time (hours), median (IQR)	23.87 (19.65–25.92)	6.47 (5.83–7.19)	6.01 (5.34–6.58)	18.742	<0.001
Appointment adherence rate (%)	76.38	84.17^#^	91.35^#^	12.856	<0.001
12-month retention rate, *n* (%) [95% CI]	86 (52.12) [44.50–59.74]	118 (73.29) [66.18–80.40]^#^	139 (85.80) [80.08–91.52]^#^^&^	45.440	<0.001
Early discontinuation rate (within 6 months), *n* (%) [95% CI]	48 (29.09) [22.18–35.99]	28 (17.39) [11.52–23.26]^#^	16 (9.88) [5.29–14.48]^#^^&^	20.060	<0.001
BCVA change (logMAR), median (IQR)	−0.08 (−0.15, −0.03)	−0.12 (−0.21, −0.06)^#^	−0.17 (−0.28, −0.09)^#^^&^	16.245	<0.001
Central retinal thickness reduction (μm), median (IQR)	57.83 (31.74–88.56)	86.92 (53.67–123.84)^#^	111.75 (77.93–155.72)^#^^&^	22.367	<0.001

^#^indicates P <0.05 compared to the historical control group;

^&^ indicates P <0.05 between the one-stop standard group and the AI-enhanced group. IQR, interquartile range; SD, standard deviation; BCVA, best-corrected visual acuity; 95% CI, 95% confidence interval. Normality was assessed using Shapiro-Wilk test. Appointment adherence rate was normally distributed (P = 0.127) and compared using one-way ANOVA with Bonferroni post-hoc test. Clinic-to-injection time, BCVA change, and central retinal thickness reduction were non-normally distributed (P <0.05) and compared using Kruskal-Wallis H test with Dunn's post-hoc test; median with interquartile range (IQR) was reported as appropriate for skewed distributions. Categorical variables (retention rate, early discontinuation rate) were compared using chi-square test; 95% CIs for proportions were calculated using Wilson method.

### 3.3 Relationship between psychosocial factors and adherence

#### 3.3.1 Changes in anxiety and depression levels

**Figure 2:**
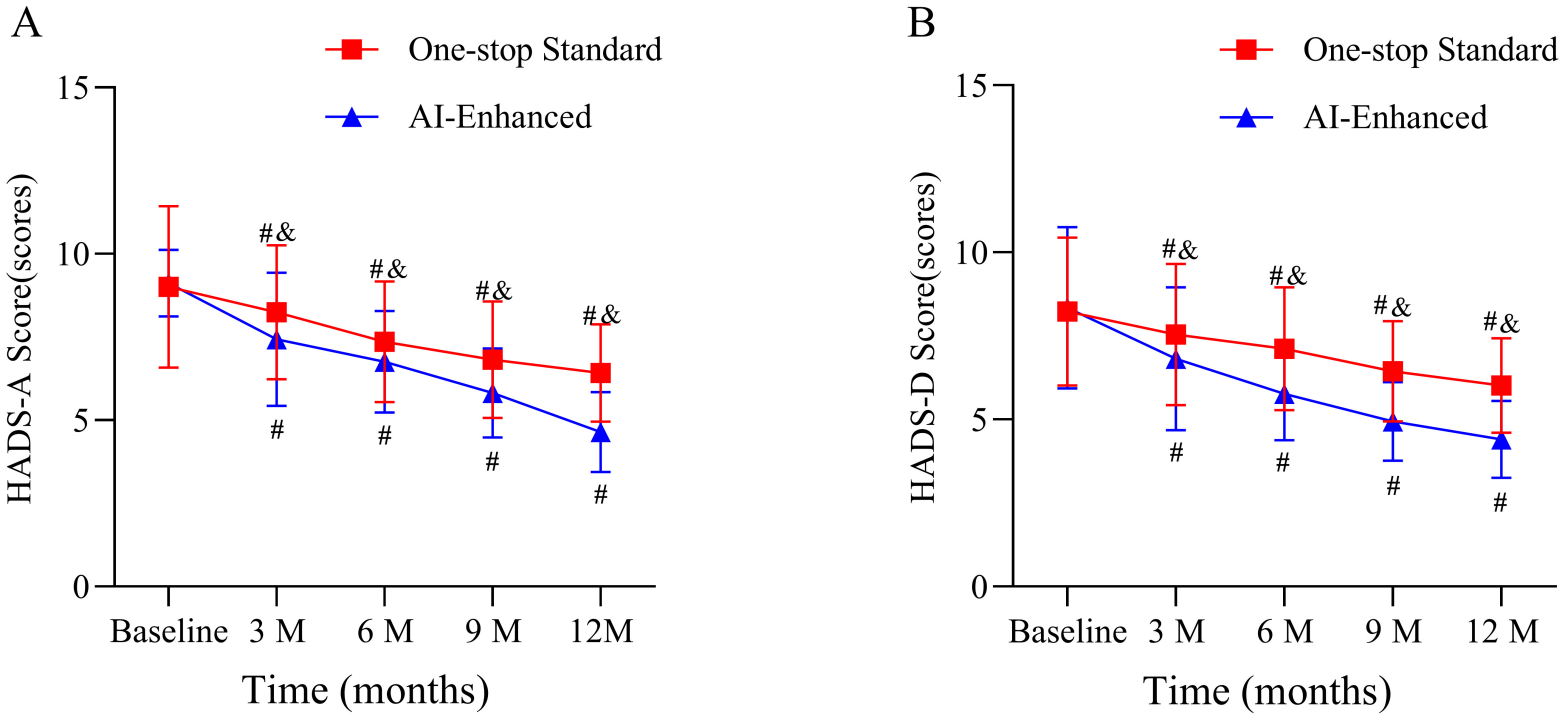
Changes in hospital anxiety and depression scale (HADS) scores over 12 months in the one-stop standard and AI-enhanced groups. **(A)** HADS-Anxiety (HADS-A) subscale scores. **(B)** HADS-Depression (HADS-D) subscale scores. ^#^ indicates *P* < 0.05 compared to baseline within the same group; ^&^ indicates *P* < 0.05 between the one-stop standard group and the AI-enhanced group at the same time point.

Psychological status in the one-stop standard and AI-enhanced groups showed significant improvement trends over time. Both groups had similar anxiety and depression scores at baseline (*P* > 0.05), but the AI-enhanced group showed more pronounced psychological status improvement during treatment. At 12 months, the AI-enhanced group had significantly lower HADS-A anxiety scores compared to the one-stop standard group (4.87 ± 1.23 vs. 6.18 ± 1.59, *P* < 0.05). Figure 2 shows the trend of changes in the psychological state of patients in the one-stop standard group and the AI-enhanced group.

### 3.4 Geospatial analysis

Geographic distance had a significant impact on treatment adherence. Stratified analysis based on the study population's median distance (38 km) showed that patients farther from the medical center (>38 km) had lower 12-month retention rates across all treatment models compared to patients living closer. However, the one-stop service model and AI-enhanced intervention significantly mitigated the negative impact of geographic distance on adherence. Table 3 shows the comparison of 12-month retention rates among different geographical distance groups.

**Table 3:** Impact of geographic distance on adherence.

Distance group	Historical control group	One-stop standard group	AI-enhanced group	Test statistic	*P*-value
≤ 38 km					
Patient number (%)	83 (50.30)	81 (50.31)	82 (50.62)	0.004	0.998
12-month retention rate, *n* (%)	51 (30.91) [23.88–37.94]	68 (42.24) [34.61–49.87]^#^	71 (43.83) [36.20–51.46]^#^	6.839	0.033
>**38 km**					
Patient number (%)	82 (49.70)	80 (49.69)^#^	80 (49.38)^#^	0.004	0.998
12-month retention rate, *n* (%)	35 (21.21) [14.97–27.45]	50 (31.06) [23.91–38.21]^#^	68 (41.98) [34.37–49.59]^#^^&^	16.380	<0.001

^#^indicates P <0.05 compared to the historical control group;

^&^ indicates P <0.05 between the one-stop standard group and the AI-enhanced group. 95% CI, 95% confidence interval. Patient distribution was compared using chi-square test. Retention rates were compared using chi-square test with Bonferroni correction for multiple comparisons. 95% CIs for proportions were calculated using Wilson method.

### 3.5 Adherence predictor analysis

#### 3.5.1 Univariate analysis

Univariate logistic regression analysis showed that lower education level ( ≤ primary school), distant residence (>38 km), poor baseline vision (>0.70 logMAR), high baseline anxiety levels (HADS-A ≥ 8), and depressive symptoms (HADS-D ≥ 8) were all significant risk factors for early treatment discontinuation. In contrast, the one-stop service model, particularly AI-enhanced intervention, significantly reduced early discontinuation risk. Table 4 shows the results of univariate predictor analysis for early discontinuation.

**Table 4:** Univariate logistic regression analysis of predictors for early treatment discontinuation (within 6 months).

Predictors	OR (95% CI)	*Z* value	*P*-value
Demographic factors			
Age (years)	1.34 (0.89–2.01)	1.402	0.162
Female	0.87 (0.58–1.31)	0.661	0.508
Education level ≤ primary school	1.89 (1.08–3.31)	2.236	0.026
Residential distance >38 km	1.76 (1.12–2.77)	2.465	0.014
Medical insurance type (rural cooperative/urban-rural resident vs. urban employee)	1.23 (0.79–1.91)	0.967	0.334
Clinical factors			
Baseline BCVA >0.70 logMAR	1.76 (1.17–2.65)	2.698	0.007
Central retinal thickness >400 μm	1.15 (0.76–1.73)	0.682	0.495
Bilateral disease	1.43 (0.89–2.30)	1.472	0.140
Baseline psychosocial factors^*^			
Baseline HADS-A ≥8 (anxiety)	3.21 (1.98–5.20)	4.576	<0.001
Baseline HADS-D ≥8 (depression)	2.14 (1.29–3.55)	2.985	0.003
Service models			
One-stop standard group vs. historical control	0.52 (0.31–0.88)	2.431	0.015
AI-enhanced group vs. historical control	0.29 (0.16–0.52)	4.287	<0.001
AI-enhanced group vs. one-stop standard group	0.56 (0.31–1.01)	1.912	0.056

This table presents univariate analysis of individual predictors. For service model comparisons, reference groups are specified in each row. OR, odds ratio; CI, confidence interval; HADS-A, hospital anxiety and depression scale-anxiety subscale; HADS-D, hospital anxiety and depression scale-depression subscale.

^*^Psychosocial factors were assessed only in the prospective cohort (one-stop standard and AI-enhanced groups).

#### 3.5.2 Multivariate analysis

Multivariate logistic regression analysis further confirmed independent predictors of early treatment discontinuation. After adjusting for other variables, residential distance >38 km (adjusted OR = 2.01), baseline high anxiety levels (adjusted OR = 2.89), and baseline HADS-D ≥8 (depression; adjusted OR = 2.31) remained significant risk factors for treatment discontinuation. One-stop and AI-enhanced intervention showed strong protective effects compared to the historical control group. Table 5 shows the results of multivariate analysis.

**Table 5:** Multivariate logistic regression analysis of early discontinuation.

Predictors	Adjusted OR (95% CI)	*Z* value	*P*-value
Residential distance >38 km	2.01 (1.24–3.26)	2.834	0.005
Baseline HADS-A ≥8 (high anxiety)	2.89 (1.68–4.97)	3.672	<0.001
Baseline HADS-D ≥8 (depression)	2.31 (1.34–3.98)	2.951	0.003
AI-enhanced group vs. historical control	0.34 (0.18–0.64)	3.286	0.001
One-stop standard group vs. historical control	0.58 (0.33–1.02)	2.093	0.038
AI-enhanced group vs. one-stop standard group	0.59 (0.32–1.08)	1.632	0.103

The multivariate model included all variables with P <0.1 in univariate analysis. OR, odds ratio; CI, confidence interval; HADS-A, hospital anxiety and depression scale-anxiety subscale; HADS-D, hospital anxiety and depression scale-depression subscale. Reference groups for service model comparisons are specified in each row. Model goodness-of-fit: C-index = 0.781, Hosmer-Lemeshow test P = 0.426.

### 3.6 Qualitative research results

A total of 42 patients completed in-depth interviews (22 from the one-stop standard group and 20 from the AI-enhanced group). Through in-depth interviews with patients in the one-stop standard group and the AI-enhanced group, five main themes were identified. Patients generally reported that the one-stop service model greatly improved healthcare convenience, and AI-enhanced intervention effectively improved treatment expectation management. Qualitative analysis supported quantitative research findings, providing important patient perspectives for service model optimization. Table 6 shows the main themes and representative quotes from qualitative analysis.

**Table 6:** Main themes and representative quotes from qualitative analysis.

Theme	Sub-theme	Representative quotes
Treatment anxiety	Procedural fear	“Every time I think about injections in my eyes, I get so nervous I can't sleep”
	Efficacy concerns	“I don't know if this treatment really works, spending so much money”
Service experience	Waiting time	“Before, I had to stay in hospital for a day, now I can go home in half a day, much more convenient”
	Doctor-patient communication	“Doctors now use tablets to show us pictures, explaining very clearly”
Expectation management	Unrealistic expectations	“I thought one or two injections would cure me, didn't expect it to take so long”
	Expectation adjustment	“The AI system made me understand this is a chronic disease requiring long-term treatment”
Social support	Family support	“Family understanding and companionship are very important for me to persist with treatment”
	Peer influence	“Seeing other patients persevere gives me confidence too”
Service improvement	Personalized needs	“Hope to arrange different times according to our situations”
	Technology application	“Mobile phone reminders are very useful, won't forget follow-up appointments”

Themes and sub-themes were identified through thematic analysis following Braun and Clarke's six-phase framework. Representative quotes were translated from Chinese and selected to illustrate key findings.

### 3.7 Safety analysis

Safety event incidence rates were low and showed no significant differences between groups (*P* > 0.05), indicating that changes in service models and introduction of AI intervention did not increase any safety risks. The most serious complication, endophthalmitis, occurred in only 1 case (0.61%) in the historical control group, with similar incidence rates of elevated intraocular pressure, subconjunctival hemorrhage, and ocular pain across groups. Table 7 shows detailed comparison of safety events among the three groups.

**Table 7:** Safety event comparison.

Adverse events	Historical control group (*n* = 165)	One-stop standard group (*n* = 161)	AI-enhanced group (*n* = 162)	Test statistic	*P*-value
Endophthalmitis, *n* (%)	1 (0.61)	0 (0.00)	0 (0.00)	1.962	0.375
Intraocular pressure elevation >21 mmHg, *n* (%)	8 (4.85)	6 (3.73)	5 (3.09)	0.696	0.706
Subconjunctival hemorrhage, *n* (%)	15 (9.10)	10 (6.21)	10 (6.17)	1.379	0.502
Ocular pain, *n* (%)	22 (13.33)	19 (11.80)	18 (11.11)	0.399	0.819

Data are presented as n (%). Adverse events were compared using chi-square test or Fisher's exact test as appropriate.

## 4 Discussion

This study systematically evaluated the impact of one-stop intravitreal injection service models and AI-enhanced interventions on anti-VEGF treatment adherence in nAMD patients through a historical control mixed-methods design. Results showed that the one-stop AI-enhanced model significantly outperformed the other two models across multiple key indicators. This finding not only provides important evidence for optimizing healthcare service processes but also reveals the important role of psychosocial factors in treatment adherence.

In service model effectiveness comparison, the one-stop AI-enhanced model performed best across key indicators including clinic-to-injection time, appointment adherence rate, 12-month retention rate, early discontinuation rate, BCVA change, and central retinal thickness reduction. The significant reduction in clinic-to-treatment time represents more efficient medical resource allocation. Particularly in chronic disease treatment environments requiring frequent visits, reducing waiting time and redundant processes is crucial for maintaining treatment motivation (23). Research shows that AMD patients require long-term anti-VEGF treatment with frequent hospital visits, and long waiting times with complex procedures may cause patient fatigue and anxiety, reducing treatment enthusiasm (24). The significant reduction in clinic-to-injection time in the AI-enhanced group helps reduce patient anxiety and fatigue caused by long waiting times, thus helping maintain patient treatment motivation.

Simultaneously, the AI-enhanced model also demonstrated advantages in improving appointment adherence rates and reducing early discontinuation. Through AI system dynamic identification of individual risk and reminder notifications, patients could establish more stable behavioral patterns during initial treatment phases, avoiding dropout due to cognitive bias, time conflicts, or treatment fatigue. Notably, the historical control group had a 12-month retention rate of 52.12%. This result is similar to Polat et al.'s study (18), which found that nAMD patients receiving ranibizumab treatment had a 51.8% treatment completion rate during 1-year follow-up. In this study, the one-stop service model improved 12-month retention rates to 73.29%, with AI-enhanced intervention further increasing to 85.80%. This result indicates that AI technology application not only improved treatment efficiency but also significantly improved patients' long-term adherence.

The negative impact of anxiety and depression on patient treatment adherence has been confirmed in multiple studies (25–27). This study further explored the relationship between anxiety and depression levels and treatment adherence through prospective cohort baseline psychosocial assessment. Results showed that patients with high anxiety levels (HADS-A ≥ 8) and high depression levels (HADS-D ≥ 8) at baseline had significantly increased early discontinuation risk. This finding reveals the dual psychological burden faced by nAMD patients: fear and anxiety about repeated intraocular injection procedures, and depressive emotions about vision loss and disease prognosis (28, 29). Notably, AI-enhanced intervention showed excellent performance in alleviating these psychological problems. At 12-month follow-up, AI-enhanced group patients had significantly lower anxiety scores compared to the standard group, with similar improvement trends in depression scores. This continuous improvement in psychological status may be an important mechanism for adherence improvement, also suggesting the necessity of integrating psychological interventions in chronic disease management.

The impact of geospatial barriers on adherence has been confirmed. A 5-year follow-up study of nAMD patients showed that 51.7% of patients stopped follow-up due to long distances from home to hospital (30). Additionally, Yan et al. (31) found that each additional 10 kilometers of Euclidean distance reduced the likelihood of patients visiting within the past quarter by 13%. Our study showed that patients residing more than 38 kilometers away had double the early discontinuation risk, further emphasizing the important impact of geospatial barriers on patient treatment adherence. Notably, one-stop service and AI intervention could partially mitigate the negative impact of geographic barriers. Among distant patients (>38 km) in the AI-enhanced group, 68 of 80 (85.0%) were retained at 12 months, compared with only 35 of 82 (42.7%) in the historical control group. This improvement stems not only from reduced visit time due to process compression but also from the AI system's scheduling module's dynamic identification and allocation of patient transportation, weather, and past behavioral patterns. This finding has important implications for medical environments with uneven resource distribution, indicating that through technological means and process innovation, the coverage of high-quality medical services can be expanded.

AI applications in healthcare have made significant progress in recent years, particularly showing great potential in disease prediction, personalized treatment, and patient management (32). AI technology can help doctors quickly and accurately diagnose diseases, formulate treatment plans, and provide personalized medical services through data analysis, machine learning, and natural language processing. In chronic disease management, AI similarly demonstrates unique advantages, predicting disease progression and adjusting treatment plans through patients' historical data and real-time monitoring, thereby improving patient treatment adherence (33, 34). In this study, the real-time expectation correction module achieved personalized patient education through natural language processing and machine learning algorithms, making this precise intervention more effective than traditional unified education. The adaptive scheduling algorithm considered patients' individual characteristics, historical behaviors, and external factors to optimize appointment arrangements and reduce no-show rates. Community-based pre-treatment counseling expanded the boundaries of medical services by training community health workers to begin intervention before patients' formal visits. This proactive intervention strategy significantly improved treatment readiness. The synergistic effect of these three components produced cumulative benefits, effectively improving nAMD patients' treatment adherence.

The clinical outcomes of this study were equally encouraging. Adherence improvement directly translated to better visual function outcomes, with AI-enhanced group patients showing significantly superior BCVA improvement and central retinal thickness reduction compared to other groups. This confirmed the direct correlation between adherence and clinical efficacy, validating the clinical value of our intervention strategies. Importantly, changes in service models and introduction of AI technology did not increase any safety risks, with similar adverse event rates across all groups, providing safety assurance for clinical implementation.

Qualitative research provided important supplementation for understanding patient experiences. Patients highly praised the convenience of one-stop service, and acceptance of AI systems exceeded expectations. The alleviation of treatment anxiety, improvement in expectation management, and enhancement of social support—these themes were highly consistent with quantitative analysis results, further validating the credibility of our findings. Patients' positive feedback on personalized services and technology applications provided direction for future service optimization.

This study has some limitations that need discussion. All patients in this study initially received ranibizumab as the anti-VEGF agent, though subsequent switching was not systematically tracked during follow-up. However, this does not affect our primary conclusions. Early discontinuation was defined as cessation within 6 months, before the typical clinical timeframe for switching decisions, which usually occurs after at least 6 months of treatment following completion of the loading phase and adequate response assessment. Furthermore, our primary outcomes—retention rate and appointment adherence rate—measure patient engagement behavior rather than drug-specific efficacy. We acknowledge this as a limitation for interpreting secondary efficacy outcomes (BCVA, central retinal thickness), though the improved clinical results are most parsimoniously explained by superior adherence rather than pharmacological differences. Future studies could consider comparing adherence patterns across different anti-VEGF agents. The historical control design of this study has inherent limitations that warrant careful consideration. Beyond the temporal trend confounding acknowledged previously, several additional factors may have differed across study periods. Staff experience and workflow maturity may have evolved, as clinical teams likely gained proficiency in patient communication and injection procedures over time, independent of the service model changes. Secular trends in patient education could also play a role, since general awareness of nAMD and anti-VEGF therapy may have improved through media coverage and community health initiatives during the study period. Healthcare system evolution, including changes in insurance policies, referral patterns, or regional healthcare infrastructure between 2022 and 2024, could have influenced adherence independently. Additionally, learning curve effects cannot be excluded, as the one-stop model itself may have undergone iterative refinements after initial implementation. Although we attempted to control through propensity score matching, unmeasured confounding factors cannot be completely excluded. Future multicenter randomized controlled trials with concurrent control groups would provide more robust evidence for causal inference.

This study did not include a formal cost-effectiveness analysis, which represents an important limitation. While the one-stop model reduces hospitalization time from approximately 24 to 6–7 h—potentially decreasing bed occupancy costs, patient transportation expenses, and caregiver time burden—and the AI-enhanced intervention may require initial investment in technology infrastructure and staff training, a comprehensive economic evaluation incorporating direct medical costs, indirect costs, and quality-adjusted life years (QALYs) is warranted. Future studies should conduct formal cost-effectiveness or cost-utility analyses to inform healthcare policy decisions and resource allocation for chronic ophthalmic disease management. Additionally, the study was conducted at a single medical center, and the generalizability of results needs further validation in different healthcare environments. The 12-month follow-up period may be insufficient to fully assess long-term adherence effects, requiring longer-term tracking studies.

Despite these limitations, this study provides important practical guidance and theoretical contributions. Research results support implementing one-stop service models in nAMD management and considering introduction of AI-enhanced interventions. The importance of psychosocial factors suggests that clinicians should incorporate adherence management into routine treatment processes, with particular attention to special needs of high-anxiety patients and distant patients. For healthcare policymakers, research results indicate that investment in service optimization and technological innovation can significantly improve chronic disease management effects, with good social benefit prospects.

## 5 Conclusion

In conclusion, this study confirmed the significant effects of one-stop intravitreal injection service models and AI-enhanced interventions in improving anti-VEGF treatment adherence in nAMD patients, identified key psychosocial determinants, and provided new insights and evidence support for precision management of chronic eye diseases. These findings not only have direct guiding significance for ophthalmic clinical practice but also provide experiences that can be applied to management of other chronic diseases requiring long-term repetitive treatment.

## Data Availability

The original contributions presented in the study are included in the article/supplementary material, further inquiries can be directed to the corresponding author.

## References

[1] Ricci F Bandello F Navarra P Staurenghi G Stumpp M Zarbin M. Neovascular age-related macular degeneration: therapeutic management and new-upcoming approaches. Int J Mol Sci. (2020) 21:8242. doi: 10.3390/ijms21218242 33153227 PMC7662479

[2] Keenan TDL Cukras CA Chew EY. Age-related macular degeneration: epidemiology and clinical aspects. Adv Exp Med Biol. (2021) 1256:1–31. doi: 10.1007/978-3-030-66014-7_1 33847996 PMC13400192

[3] Tricco AC Thomas SM Lillie E Veroniki AA Hamid JS Pham B . Anti-vascular endothelial growth factor therapy for age-related macular degeneration: a systematic review and network meta-analysis. Syst Rev. (2021) 10:315. doi: 10.1186/s13643-021-01864-6 34930439 PMC8690960

[4] Nguyen CL Oh LJ Wong E Wei J Chilov M. Anti-vascular endothelial growth factor for neovascular age-related macular degeneration: a meta-analysis of randomized controlled trials. BMC Ophthalmol. (2018) 18:130. doi: 10.1186/s12886-018-0785-3 29843663 PMC5975529

[5] Cheema MR DaCosta J Talks J. Ten-year real-world outcomes of anti-vascular endothelial growth factor therapy in neovascular age-related macular degeneration. Clin Ophthalmol. (2021) 15:279–87. doi: 10.2147/OPTH.S269162 33519189 PMC7837532

[6] Holekamp N Gentile B Giocanti-Aurégan A García-Layana A Peto T Viola F . Patient experience survey of anti-vascular endothelial growth factor treatment for neovascular age-related macular degeneration and diabetic macular edema. Ophthalmic Res. (2024) 67:311–21. doi: 10.1159/000540390 38679018

[7] Matonti F Korobelnik JF Dot C Gualino V Soler V Mrejen S . Comparative effectiveness of intravitreal anti-vascular endothelial growth factor therapies for managing neovascular age-related macular degeneration: a meta-analysis. J Clin Med. (2022) 11:1834. doi: 10.3390/jcm11071834 35407439 PMC8999505

[8] Vogt D Deiters V Herold TR Guenther SR Kortuem KU Priglinger SG . Optimal patient adherence and long-term treatment outcomes of neovascular age-related macular degeneration in real-life. Curr Eye Res. (2022) 47:889–96. doi: 10.1080/02713683.2022.2044056 35179427

[9] Koizumi H Gomi F Tsujikawa A Honda S Mori R Ochi H . Efficacy, durability, and safety of faricimab up to every 16 weeks in patients with neovascular age-related macular degeneration: 2-year results from the Japan subgroup of the phase III TENAYA trial. Graefes Arch Clin Exp Ophthalmol. (2024) 262:2439–48. doi: 10.1007/s00417-024-06377-1 38483611 PMC11271316

[10] Nowak M Cybulska AM Schneider-Matyka D Grochans E Walaszek I Panczyk M . Acceptance of the disease in patients diagnosed with neovascular age-related macular degeneration depending on visual parameters-before and after a series of seven intravitreal injections. J Clin Med. (2025) 14:447. doi: 10.3390/jcm14020447 39860453 PMC11765872

[11] Wolfram C Pfeiffer N Hudde T Klatt A Schnegelsberg B Ross M . The psychological, social and behavioral impact of intravitreal anti-vegf therapy: an analysis from the ALBATROS data. J Clin Med. (2023) 12:7435. doi: 10.3390/jcm12237435 38068487 PMC10707522

[12] Sii S Aspinall P Borooah S Dhillon B. Exploring factors predicting changes in patients' expectations and psychosocial issues during the course of treatment with intravitreal injections for wet age-related macular degeneration. Eye. (2018) 32:673–8. doi: 10.1038/eye.2017.271 29219960 PMC5898856

[13] Shirian JD Shukla P Singh RP. Exploring new horizons in neovascular age-related macular degeneration: novel mechanisms of action and future therapeutic avenues. Eye. (2025) 39:40–4. doi: 10.1038/s41433-024-03373-x 39379521 PMC11733175

[14] Meer EA Oh DH Brodie FL. Time and distance cost of longer acting anti-VEGF therapies for macular degeneration: contributions to drug cost comparisons. Clin Ophthalmol. (2022) 16:4273–9. doi: 10.2147/OPTH.S384995 36578665 PMC9792116

[15] Ramakrishnan MS Yu Y VanderBeek BL. Association of visit adherence and visual acuity in patients with neovascular age-related macular degeneration: secondary analysis of the comparison of age-related macular degeneration treatment trial. JAMA Ophthalmol. (2020) 138:237–42. doi: 10.1001/jamaophthalmol.2019.4577 32027349 PMC7042935

[16] Tan L Ma Z Miao Q Liu S Li Y Ke Y . Knowledge, attitudes, and practices of patients with age-related macular degeneration (AMD) towards anti-VEGF treatment under one-stop intravitreal injection model. Sci Rep. (2024) 14:26563. doi: 10.1038/s41598-024-77999-y 39496825 PMC11535370

[17] Sumner J Bundele A Lim HW Phan P Motani M Mukhopadhyay A. Developing an artificial intelligence-driven nudge intervention to improve medication adherence: a human-centred design approach. J Med Syst. (2023) 48:3. doi: 10.1007/s10916-023-02024-0 38063940 PMC10709244

[18] Polat O Inan S Özcan S Dogan M Küsbeci T Yavaş GF . Factors affecting compliance to intravitreal anti-vascular endothelial growth factor therapy in patients with age-related macular degeneration. Turk J Ophthalmol. (2017) 47:205–10. doi: 10.4274/tjo.28003 28845324 PMC5563548

[19] Association WM. World medical association declaration of helsinki: ethical principles for medical research involving human subjects. JAMA. (2013) 310:2191–4. doi: 10.1001/jama.2013.281053 24141714

[20] Kaiser PK Wykoff CC Singh RP Khanani AM Do DV Patel H . Retinal fluid and thickness as measures of disease activity in neovascular age-related macular degeneration. Retina. (2021) 41:1579–86. doi: 10.1097/IAE.0000000000003194 33949342 PMC8297539

[21] Zigmond AS Snaith RP. The hospital anxiety and depression scale. Acta Psychiatr Scand. (1983) 67:361–70. doi: 10.1111/j.1600-0447.1983.tb09716.x 6880820

[22] Xie E Wong SC Bai Y. Using Nvivo to analyze the impact of computer simulation of parent-child cooperative art activities on the growth of preschool children. J Autism Dev Disord. (2024) 54:4195–207. doi: 10.1007/s10803-023-06124-1 37713171

[23] Moura A Pinho M. A scheduling optimization approach to reduce outpatient waiting times for specialists. Healthcare. (2025) 13:749. doi: 10.3390/healthcare13070749 40218047 PMC11988311

[24] Thier A Breuning M Wolfram C Zeitz O Holmberg C. Emotional and physical experiences of people with neovascular age-related macular degeneration during the injection process in Germany: a qualitative study. BMJ Open. (2022) 12:e058266. doi: 10.1136/bmjopen-2021-058266 35705348 PMC9204446

[25] Volpato E Toniolo S Pagnini F Banfi P. The relationship between anxiety, depression and treatment adherence in chronic obstructive pulmonary disease: a systematic review. Int J Chron Obstruct Pulmon Dis. (2021) 16:2001–21. doi: 10.2147/COPD.S313841 34262270 PMC8275112

[26] Poletti V Pagnini F Banfi P Volpato E. The role of depression on treatment adherence in patients with heart failure-a systematic review of the literature. Curr Cardiol Rep. (2022) 24:1995–2008. doi: 10.1007/s11886-022-01815-0 36327056 PMC9747824

[27] Davis NE Hue JJ Kyasaram RK Elshami M Graor HJ Zarei M . Prodromal depression and anxiety are associated with worse treatment compliance and survival among patients with pancreatic cancer. Psychooncology. (2022) 31:1390–8. doi: 10.1002/pon.5945 35470512

[28] Lee CY Chen HC Huang JY Lai CC Lin HY Yang SF . Increased probability of mood disorders after age-related macular degeneration: a population-based cohort study. Sci Rep. (2022) 12:15222. doi: 10.1038/s41598-022-19429-5 36075924 PMC9458640

[29] Robinson K Cooper SJ Persaud S Frederick JL Singh RP. Discordance among patients and ophthalmologists regarding the burden of intravitreal injections. Clin Ophthalmol. (2025) 19:2637–45. doi: 10.2147/OPTH.S532179 40800716 PMC12341839

[30] Boulanger-Scemama E Querques G About F Puche N Srour M Mane V . Ranibizumab for exudative age-related macular degeneration: a five year study of adherence to follow-up in a real-life setting. J Fr Ophtalmol. (2015) 38:620–7. doi: 10.1016/j.jfo.2014.11.015 25913443

[31] Yan LD Pierre-Louis D Isaac BD Jean-Baptiste W Vertilus S Fenelon D . Does distance from a clinic and poverty impact visit adherence for noncommunicable diseases? A retrospective cohort study using electronic medical records in rural Haiti. BMC Public Health. (2020) 20:1545. doi: 10.1186/s12889-020-09652-y 33054756 PMC7556963

[32] Alhumaidi NH Dermawan D Kamaruzaman HF Alotaiq N. The use of machine learning for analyzing real-world data in disease prediction and management: systematic review. JMIR Med Inform. (2025) 13:e68898. doi: 10.2196/68898 40537090 PMC12226786

[33] Thorat V Rao P Joshi N Talreja P Shetty AR. Role of artificial intelligence (AI) in patient education and communication in dentistry. Cureus. (2024) 16:e59799. doi: 10.7759/cureus.59799 38846249 PMC11155216

[34] Soellner M Koenigstorfer J. Compliance with medical recommendations depending on the use of artificial intelligence as a diagnostic method. BMC Med Inform Decis Mak. (2021) 21:236. doi: 10.1186/s12911-021-01596-6 34362359 PMC8344186

